# Long-term consequences of one anastomosis gastric bypass on esogastric mucosa in a preclinical rat model

**DOI:** 10.1038/s41598-020-64425-2

**Published:** 2020-04-30

**Authors:** Matthieu Siebert, Lara Ribeiro-Parenti, Nicholas D. Nguyen, Muriel Hourseau, Belinda Duchêne, Lydie Humbert, Nicolas Jonckheere, Grégory Nuel, Jean-Marc Chevallier, Henri Duboc, Dominique Rainteau, Simon Msika, Nathalie Kapel, Anne Couvelard, André Bado, Maude Le Gall

**Affiliations:** 1 Inserm UMRS 1149, UFR de Médecine Paris Diderot, Université de Paris, AP-HP Paris, France; 2 Department of digestive Surgery, AP-HP, Hôpital Européen Georges Pompidou, Paris, France; 3 Department of General and Visceral Surgery, AP-HP, Bichat-Claude Bernard Hospital, Paris, France; 4 Department of anatomopathology, AP-HP, Bichat-Claude Bernard Hospital, Paris, France; 5 Univ. Lille, CNRS, Inserm, CHU Lille, UMR9020 – UMR-S 1277 - Canther – Cancer Heterogeneity, Plasticity and Resistance to Therapies, Lille, France; 6 Inserm UMR 7203, AP-HP Saint Antoine hospital, Paris, France; 7 Stochastics and Biology Group (MAV), Probability and Statistics (LPSM), CNRS 8001, Sorbonne Université, Paris, France; 8 Laboratoire de Coprologie Fonctionnelle, Hôpital Pitié-Salpêtrière Charles Foix, AP-HP, Paris, France

**Keywords:** Obesity, Gastrointestinal models

## Abstract

Although bariatric surgery is proven to sustain weight loss in morbidly obese patients, long-term adverse effects have yet to be fully characterized. This study compared the long-term consequences of two common forms of bariatric surgery: one-anastomosis gastric bypass (OAGB) and Roux-en-Y Gastric Bypass (RYGB) in a preclinical rat model. We evaluated the influence of biliopancreatic limb (BPL) length, malabsorption, and bile acid (BA) reflux on esogastric mucosa. After 30 weeks of follow-up, Wistar rats operated on RYGB, OAGB with a short BPL (15 cm, OAGB-15), or a long BPL (35 cm, OAGB-35), and unoperated rats exhibit no cases of esogastric cancer, metaplasia, dysplasia, or Barrett’s esophagus. Compared to RYGB, OAGB-35 rats presented higher rate of esophagitis, fundic gastritis and perianastomotic foveolar hyperplasia. OAGB-35 rats also revealed the greatest weight loss and malabsorption. On the contrary, BA concentrations were the highest in the residual gastric pouch of OAGB-15 rats. Yet, no association could be established between the esogastric lesions and malabsorption, weight loss, or gastric bile acid concentrations. In conclusion, RYGB results in a better long-term outcome than OAGB, as chronic signs of biliary reflux or reactional gastritis were reported post-OAGB even after reducing the BPL length in a preclinical rat model.

## Introduction

Bariatric surgery is widely accepted as a long-term effective treatment for morbid obesity and ensuing metabolic disorders^1^. One anastomosis gastric bypass (OAGB) is a promising procedure, first reported in 1997^2^. This intervention has been proven safe^2^ with some studies even reporting a lower rate of post-operative morbidity compared to the gold standard, Roux-en-Y gastric bypass (RYGB)^3,4^. Additionally, OAGB may be preferred to RYGB due to its convenient ability to be reversed^5^ and revised^6^. Its efficiency in terms of weight loss and control of comorbidities has been widely characterized in the last several decades^7–9^, prompting its potential as an improved alternative to RYGB^7^.

However, OAGB is still debated due to chronic risks associated with potential biliary reflux^10^ on the esogastric tract.

OAGB, as all Omega-loop surgical strategies, is characterized by the direct anastomosis of the biliopancreatic loop to the stomach, instead of interposing an alimentary loop as in the Roux-en-Y procedures. This anatomically exposes the esogastric tract to bile acids (BA). In rats, the negative consequences of Omega-loop surgical strategies are well known^11,12^. Esojejunal or esoduodenal anastomoses have been reported as experimental models of induced esophageal carcinogenesis^12–14^. Additionally in humans, Billroth II anastomosis, used for reconstruction after gastric cancer or gastric ulcer surgery, is associated with an increased risk of esogastric metaplasia and cancer compared to Roux-en-Y reconstruction^15–17^. A physiological hypothesis for these phenomena is that BA reflux could be responsible for chronic inflammation and oxidative stress – two major factors in the initiation of esophageal intestinal metaplasia and the eventual progression to adenocarcinoma^18,19^. Accordingly the first two cases of adenocarcinoma of the esophagogastric junction (AEG) following OAGB have been recently reported; the first reported a carcinoma of the gastric cardia (AEG II)^20^ and the second reported an adenocarcinoma of the esophagus (AEG I)^21^. However, it is important to note that in both cases patients were suffering from chronic reflux before and after the surgery.

Another point of debate is the impact of nutritional status on carcinogenesis. On one hand, epidemiological studies have revealed a strong link between obesity and cancer^22^. On the other, experimental studies have demonstrated that a lack of folate^23^, magnesium^24^, or vitamin D^25^ could act as co-factors in driving human digestive carcinogenesis. Broadly speaking, it is well known that bariatric surgery, particularly OAGB, may be responsible for these deficiencies^26–28^ or undernutrition^29^.

While OAGB has been performed for over 20 years, little objective data have described its long-term effects. To our knowledge, few cases of gastric cancer have been reported after omega-loop gastric bypass, an operation following similar principles to those of OAGB. However, three of these cancers were located in the region of the stomach excluded from the alimentary tract and only one of these cancers was located in the gastric pouch^10^. As of yet, only two published cases of esophageal or gastric cancer in patients post-OAGB have been published^20,21^ but there are no published long-term endoscopic studies. We previously explored the middle-term consequences of OAGB-induced biliary reflux on rats after 16 weeks and did not report an increased risk for esogastric cancer^30^.

The objective of this study was to evaluate long-term physiological consequences of OAGB on esogastric mucosa using a validated experimental model. OAGB rats were primarily compared to the gold standard RYGB and we hypothesized that OAGB rats were at a higher risk for post-operative precancerous or cancerous lesions. In order to better characterize the potential for adverse carcinogenic side effects in the esogastric physiology, we also analyzed the impact of the OAGB biliopancreatic limb (BPL) length on mucosal inflammation, BA concentration in the gastric pouch, and malabsorption after long-term follow-up.

## Results

A schematic view of the experimental plan is presented in Supplementary Fig. S1.

### Body weight, food intake, and caloric loss after bariatric surgery in lean animals

**Figure 1:**
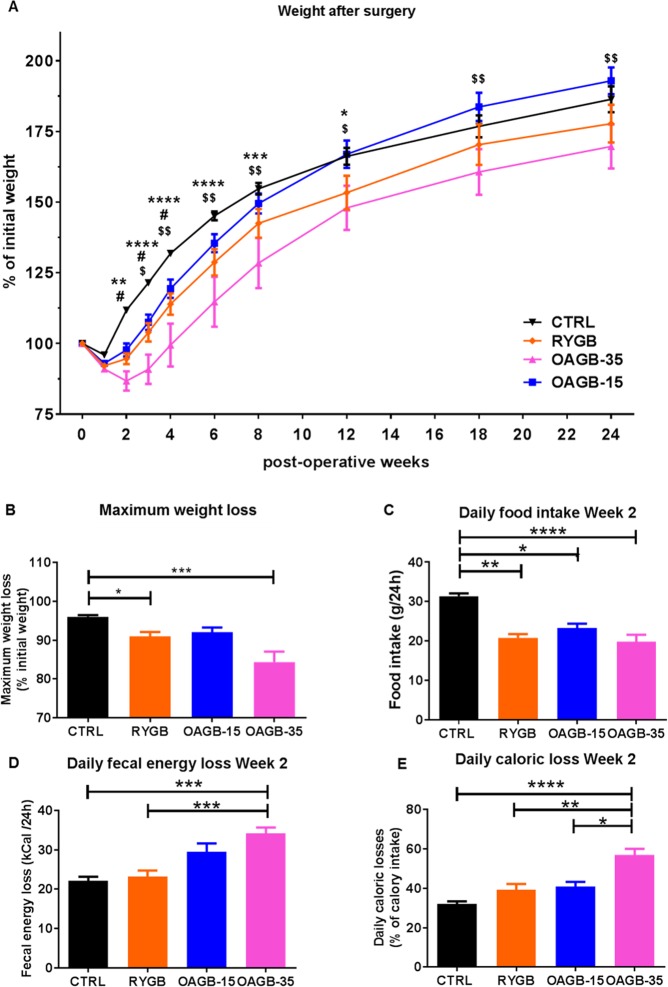
Gastric Bypass surgery in lean rats induced a delay in weight gain and OAGB-35 is associated with reduced food intake and increased malabsorption. (**A**) Weight loss and regain according to surgical procedure during 24 post-operative weeks. *P < 0.05; ***P < 0.001, ****P < 0.0001 OAGB-35 vs. unoperated CTRL. ^#^*P* < 0.05 RYGB vs. unoperated CTRL. ^$^*P* < 0.05; ^$$^*P* < 0.01 OAGB-35 vs. OAGB-15 by Tukey’ multiple comparison tests after 2-way ANOVA. (**B**) Maximum weight loss according to surgical procedure, (**C**) daily food intake, (**D**) fecal energy loss and, (**E**) caloric loss during the second postoperative week. *P < 0.05; **P < 0.01; ***P < 0.001, ****P < 0.0001 by Dunn’s multiple comparison tests after Kruskal Wallis test.

As previously observed, all bypass surgeries induced a rapid although transient weight loss, and maximal weight loss was observed between 10 and 14 days after surgery (Fig. 1A). The OAGB-35 group experienced the greatest weight loss, about 2-fold that experienced by the OAGB-15 and RYGB groups (OAGB-35 −15.6 ± 2.7% vs. OAGB-15 −7.9 ± 1.2% vs. RYGB −9.0 ± 1.1%) (Fig. 1B). As a baseline control, we compared operated rats to unoperated control (CTRL) rats, and as expected, the CTRL group experienced the lowest observed weight loss (−4 ± 0.4%) (Fig. 1B). In the operated groups, weight loss was due to both reduced food intake and increased fecal caloric losses (Fig. 1C,D). Accordingly, daily caloric losses (Fig. 1E) during the second postoperative week were statistically more important in the OAGB-35 group (56.8 ± 3.2% of calorie intake) compared to all groups, CTRL rats (32 ± 1.3%, *P* < 0.0001), RYGB (39.2 ± 2.9%, *P* < 0.001) and OAGB-15 (40.9 ± 2.4%, *P* < 0.01).

We operated on non-obese young rats and followed them for 30 weeks, finding that all rats eventually regained weight over time. However, it is important to note that operated animals presented delayed weight gain compared to unoperated controls (Fig. 1A). After 30 weeks, all groups almost doubled their weight (CTRL: 186 ± 4.6%; RYGB: 178 ± 6.7%; OAGB-15: 193 ± 4.7%; OAGB-35: 170 ± 7.7%). Rats experienced almost 12% less weight gain post OAGB-35 than post OAGB-15 (*P* < 0.05). Although not statistically significant, OAGB-35 rats experienced almost 7% less weight gain than the RYGB rats.

### Esophagus and gastric lesions after bypass surgeries

**Figure 2:**
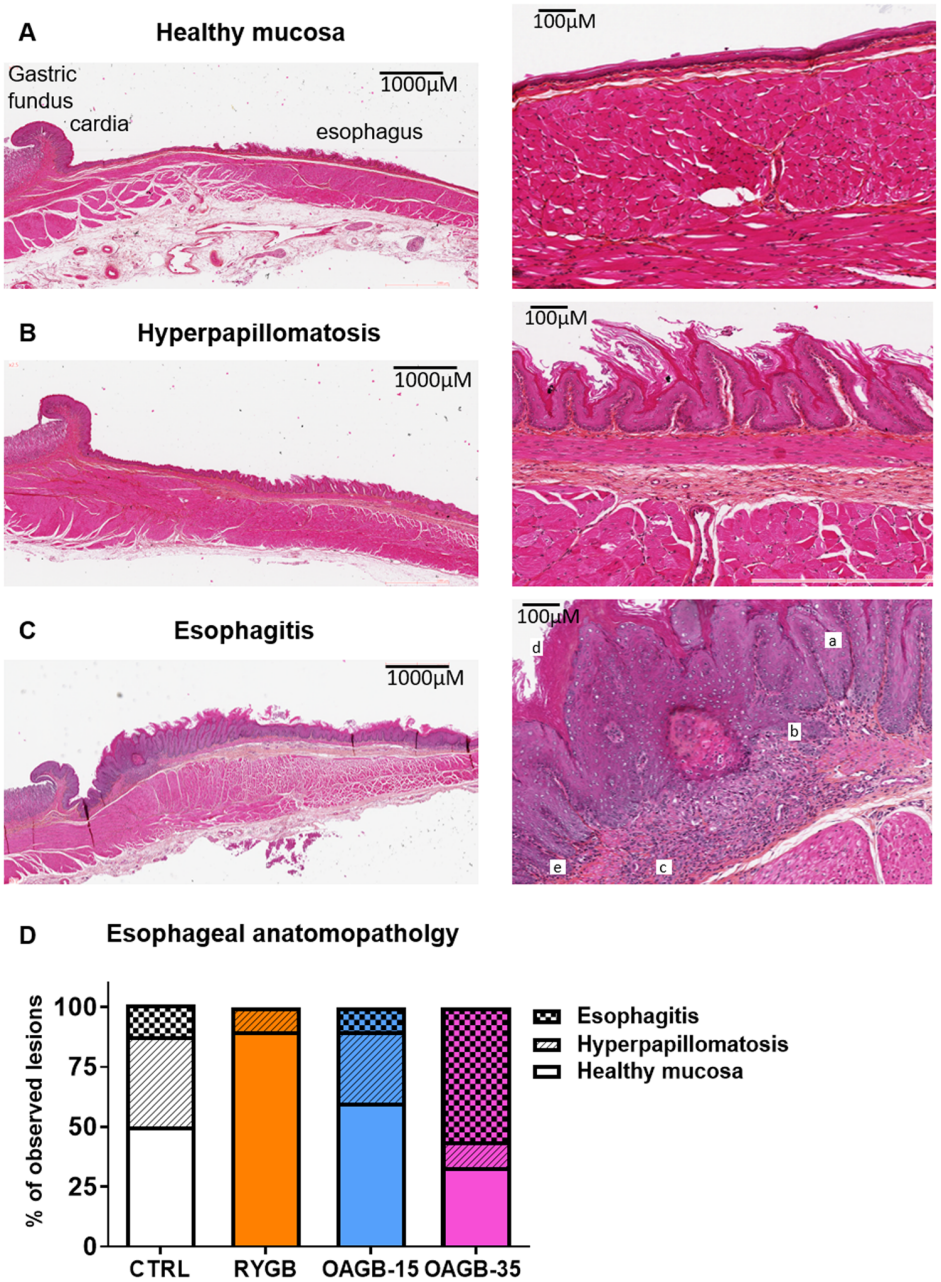
One-anastomosis gastric bypass with extended biliopancreatic limb increases risk for esogastric lesions. (**A**–**C**) Representative HES staining of healthy esophageal mucosa (**A**), esophageal hyperpapillomatosis (**B**) and esophagitis (**C**) illustrating an association with hyperpapillomatosis (a), basal cell proliferation (b), fibrosis (c), hyper ortho-keratosis (d), and immune cell infiltration (e). (**D**) Quantification of esophageal lesions according to surgical procedure and expressed as a percent. CTRL n = 8, RYGB n = 10, OAGB-15 n = 10 and OAGB-35 n = 10.

To analyze putative esophageal lesions resulting from the different surgeries, HES-staining of esophageal mucosa were analyzed (Fig. 2 and Supplementary Fig. S2) and scored as either healthy esophageal mucosa (HEM, Fig. 2A), esophageal hyperpapillomatosis (EHP, Fig. 2B), or esophagitis (Fig. 2C). Surprisingly, unoperated control animals displayed more esophageal lesions than RYGB animals (50% of CTRL, n = 4/8 vs. 10% of RYGB, n = 1/10). Specifically, EHP was observed in only 1/10 rat from the RYGB group with no occurrence of esophagitis whereas 4/8 rats in the unoperated group showed signs of EHP with 1/8 experiencing esophagitis.

Histological lesion occurrence in the OAGB-15 group was close to that of the unoperated group with 60% of the samples experiencing HEM (n = 6/10); 30% experiencing EHP (n = 3/10); and 10% experiencing esophagitis (n = 1/10). The OAGB-35 group had the highest percentage of esophagitis (50%; n = 5/10) and the lowest percentage of HEM (30%, n = 3/10) compared to the other groups.

Esophageal anatomopathology was analyzed using Fisher exact test to determine statistical significance (Supplementary Tables 1–4 and Supplementary Text 1). The percentage of observed esophageal lesions was statistically different between groups (*P* < 0.001). Rats that underwent RYGB displayed a lower percentage of esophageal lesions when compared to unoperated rats although the difference was not significant (*P* = 0.157). With the study design, the power (at level 5%) for the corresponding comparison is 0.352 suggesting that the non-significance might be due to the low power of the study. Most importantly, there was a prominent increase in the percentage of esophageal lesions present in OAGB-35 when compared to RYGB (*P* = 0.008).

**Figure 3:**
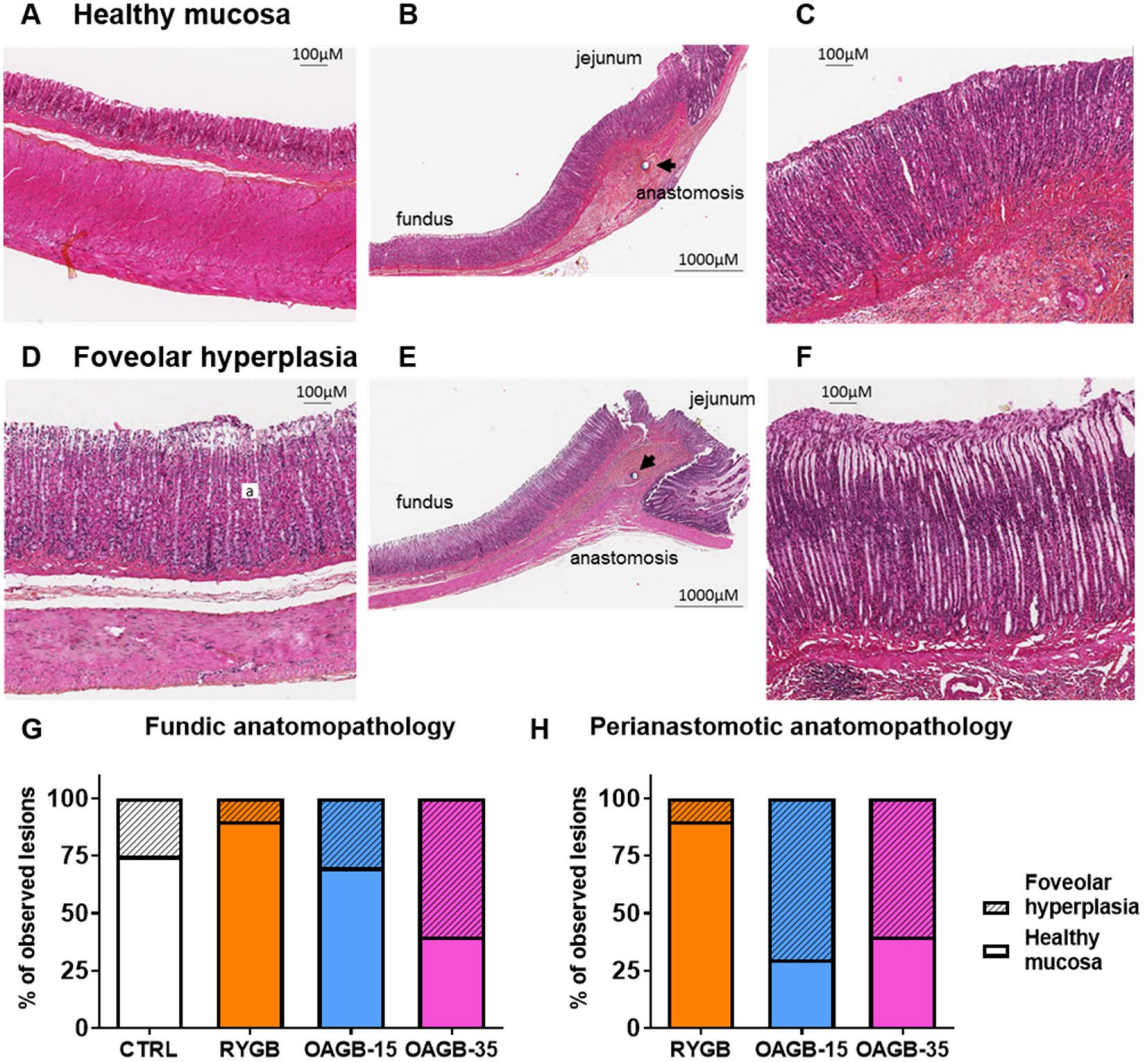
OAGB is associated with elevated perianastomotic and fundic foveolar hyperplasia. (**A**–**C**) Representative HES staining of a healthy mucosa next to a gastrojejunal anastomosis (arrow points to the anastomosis). (**D**,**E**) Representative HES staining of (**D**) fundic and (**F**) perianastomotic foveolar hyperplasia displaying parietal cell proliferation (a). (**G**,**H**) Quantification of gastric anatomopathological lesion in the fundus (**G**) and near the anastomosis (**H**) expressed as a percent. CTRL n = 8, RYGB n = 10, OAGB-15 n = 10 and OAGB-35 n = 10.

In order to evaluate changes in the fundic mucosa, we characterized the histological slices as having either healthy gastric mucosa (HGM, Fig. 3A–C) or foveolar hyperplasia (FH, Fig. 3D–F). The fundic mucosa was analyzed in all animals (Fig. 3G), and for those that underwent gastric bypass, the region of the gastro-jejunal anastomosis was also studied (Fig. 3H). No precancerous or cancerous lesions were observed.

The OAGB-35 group presented the highest number of altered mucosa with 60% (n = 6/10) FH in the fundic mucosa as in the region of the gastro-jejunal anastomosis while RYGB presented only 10% (n = 1/10) FH in both regions. Statistical analyses of fundic and perianastomotic mucosa anatomopathology (Supplementary Table 2–4 and Supplementary Text 1) revealed that the percentage of gastropathy was close to being statistically different between RYGB and OAGB-35 (*P* = 0.0573 for both fundic and perianastomotic mucosa). The corresponding power at a level of 5% is 0.436 for fundic and 0.444 for perianastomotic mucosa. However, the combination of both tables reveals a *P*-value of 0.0137 confirming the fact that lesions in the fundic and perianastomotic mucosa after OAGB-35 were more important than after RYGB.

We complemented these anatomopathological analyses by performing immunohistochemistry, probing for Mucin 2, Mucin 4 and Mucin 5B (Supplementary Fig. S2) as putative markers of Barrett’s esophagus. Compared to healthy mucosa, no additional staining was observed in hyperpapillomatosis or esophagitis lesions from either the esophagus or fundus of the stomach, suggesting the absence of Barrett’s esophagus.

### Gastric biliary acid content

**Figure 4:**
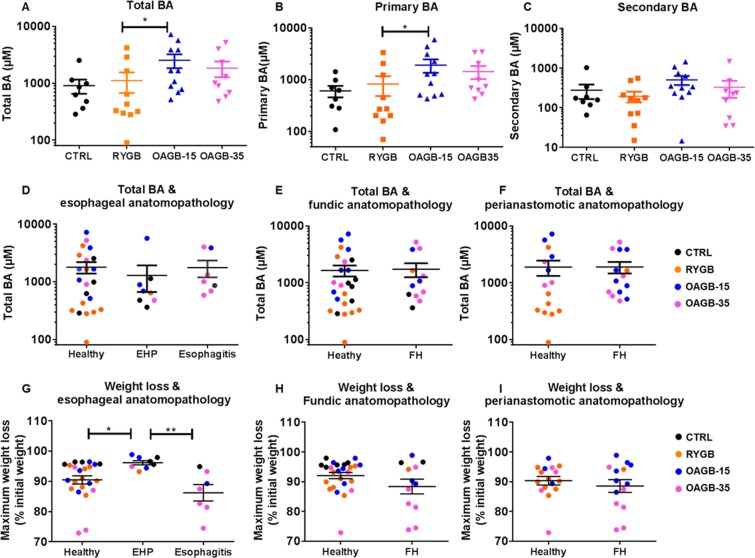
Bile acid concentrations are higher in the gastric pouch of OAGB groups but not associated with esogastric pathologies. (**A**) Total, (**B**) primary and (**C**) secondary biliary acid (BA) concentration (µM) within the stomach (CTRL) and remaining gastric pouch (post-gastric bypass) according to surgical procedure. Esophageal (**D**), fundic (**E**), and perianastomosis (**F**) anatomopathology according to BA concentrations. Esophageal (**G**), fundic (**H**) and perianastomotic (**I**) anatomopathology according to maximum weight loss. (CTRL in black, RYGB in orange, OAGB-15 in blue, OAGB-35 in pink). *P < 0.05 by Dunn’s multiple comparison tests after Kruskal Wallis test. Abbreviations: EHP: esophageal hyperpapillomatosis; FH: Foveolar Hyperplasia.

Biliary acid (BA) reflux within the gastric pouch has been a controversial post-operative long-term consequence of OAGB. To gain a better understanding of the effects of gastrointestinal modifications post-bariatric surgery, we measured total BA, primary BA, and secondary BA within the stomachs of unoperated CTRL animals and the residual gastric pouches of operated rats (Fig. 4A–C).

Detailed statistical analyses (Supplementary Table 5 and Supplementary Text 2) shows that total, primary and secondary BA concentrations were not statistically different between CTRL and RYGB animals (*P* = 0.649, *P* = 0.779, and *P* = 0.342 RYGB vs. CTRL respectively). However total and primary BA concentrations were statistically different between the three operated groups (*P* = 0.0504 and *P* = 0.0375 respectively). In particular, concentrations of total and primary BA were about 2.3 fold higher in OAGB-15 animals compared to RYGB animals (OAGB-15 = 2,536 µM vs. RYGB = 1,114 µM, *P* = 0.0279 for total BA and OAGB-15 = 1,909 µM vs. RYGB = 822 µM, *P* = 0.0271 for primary BA). No other statistical difference was highlighted; although, a trend towards increased levels of secondary BA was observed in the two OAGB groups in comparison to RYGB with *P* = 0.179 and a power at level 5% of 0.356.

To test whether gastric BA concentration is associated with esophageal or gastric lesions independent of surgical procedure, we pooled all subjects from each group and classified them based on anatomopathology (Fig. 4D–F). Considering all groups together, total BA gastric concentrations were not associated with an abnormal esophageal (*P* = 0.40), fundic (*P* = 0.59) or perianastomotic (*P* = 0.42) anatomopathology.

#### Undernutrition markers

We suspected that the OAGB-associated esogastric lesions were linked to undernutrition. To test whether weight loss was associated with abnormal esophageal anatomopathology, rats were compared according to their maximal weight loss but independent of surgical procedure. An intriguing observation was that hyperplasia was associated with a lower weight loss compared to healthy esophageal mucosa or esophagitis (Fig. 4G). The development of esophagitis on the other hand did not appear to correlate with weight loss across all groups. Similarly, weight loss was not associated with fundic or perianastomotic foveolar hyperplasia (Fig. 4H,I).

Plasma concentrations of creatinine, urea, albumin, protein, calcium, phosphates, Vitamin D, iron, ferritin, transferrin, triglycerides, total and HDL cholesterol, NEFA, ALAT, and ASAT were assayed to determine whether animals were undernourished 30 weeks after surgery (Table 1). Although OAGB-35 animals display abnormal values compared to unoperated CTRL rats for some of these markers (notably higher uremia and lower vitamin D) no association between anatomical lesions and biochemical parameters were discerned.

**Table 1:** Main biochemical parameters measured 30 weeks after surgery.

Plasma concentrations		CTRL	RYGB	OAGB 15	OAGB 35	KW P value
Creatinine	Mean	53	52.4	54.4	50.6	0.2047
(µmol/L)	SEM	1.461	1.424	1.352	0.8718	ns
	Stats		ns	ns	ns	
Urea	Mean	4.583	5.61	5.3	5.86	0.0007
(mmol/L)	SEM	0.08724	0.2942	0.09145	0.1845	***
	Stats		*	*	***	
Albumin	Mean	26.89	24.19	26.33	25.76	0.0271
(g/L)	SEM	0.8081	0.5381	0.4206	0.6318	*
	Stats		*	ns	ns	
Protein	Mean	54.01	50.73	54.9	54.65	0.0331
(g/L)	SEM	0.642	1.104	0.9747	0.9507	*
	Stats		ns	ns	ns	
Calcium	Mean	2.447	2.343	2.364	2.368	0.0674
(mmol/L)	SEM	0.01742	0.0206	0.0201	0.03524	ns
	Stats		*	ns	ns	
Phosphate	Mean	2.146	2.08	2.341	2.215	0.0159
(mmol/L)	SEM	0.07819	0.05434	0.04581	0.05689	*
	Stats		ns	ns	ns	
Vitamin D	Mean	64.95	49.4	54.87	41.51	0.0277
(nmol/L)	SEM	4.877	4.723	2.917	5.059	*
	Stats		ns	ns	**	
Iron	Mean	26.96	12.35	18.2	13.84	0.003
(µmol/L)	SEM	2.701	2.566	1.735	1.98	**
	Stats		**	ns	**	
Ferritin	Mean	51.56	36.39	49.06	41.88	0.3415
(ng/mL)	SEM	4.964	4.437	8.584	10.51	ns
	Stats		ns	ns	ns	
Transferrin	Mean	1.136	1.156	1.163	1.205	0.9743
(g/L)	SEM	0.07393	0.03458	0.03462	0.06337	ns
	Stats		ns	ns	ns	
Triglycerides	Mean	0.79	0.8444	0.7045	0.858	0.6756
(mmol/L)	SEM	0.09256	0.12	0.07453	0.0964	ns
	Stats		ns	ns	ns	
Total Cholesterol (mmol/L)	Mean	3.491	1.981	2.083	2.09	0.0037
	SEM	0.5467	0.1093	0.112	0.1482	**
	Stats		**	**	*	
HDL Cholesterol (mmol/L)	Mean	1.637	0.835	0.9873	0.986	0.0029
	SEM	0.2435	0.07046	0.07289	0.08543	**
	Stats		***	*	*	
NEFA	Mean	1.064	0.5111	0.6336	0.414	0.0605
(mmol/L)	SEM	0.2308	0.1806	0.2135	0.07317	ns
	Stats		*	ns	ns	
ALAT	Mean	45.86	52.44	64.64	56.6	0.0288
(U/L)	SEM	3.801	7.485	3.781	4.655	*
	Stats		ns	*	ns	
ASAT	Mean	117.1	98	167.8	147.4	0.0618
(U/L)	SEM	9.585	7.885	23.85	18.17	ns
	Stats		ns	ns	ns	

Mean and Standard error of mean (SEM) of plasma concentrations of creatinine (µmol/L), urea (nmol/L), albumin (g/L), protein (g/L), calcium (mmol/L), phosphates (mmol/L), vitamin D (nmol/L), iron (µmol/L), ferritin (ng/mL), transferrin (g/L), triglycerides (mmol/L), total and HDL cholesterol (mmol/L), Non esterified Fatty Acids (NEFA) (mmol/L), Alanine Aminotransferase (ALAT) (U/L), and Aspartate Aminotransferase (ASAT) (U/L).

Comparison between groups was conducted using Kruskal Wallis (KW) non-parametric tests followed by Dunn’s multiple comparisons test (Stats) to compare bypass with CTRL.

*P < 0.05; **P < 0.01***P < 0.001.

## Discussion

Following 30 weeks post-OAGB and -RYGB, we showed that no rats displayed signs of metaplasia, dysplasia, Barrett’s esophagus, or esogastric cancer. We must note however that OAGB-35 rats experienced higher rates of esophagitis in addition to fundic and perianastomotic foveolar hyperplasia compared to the RYGB group. Furthermore, although we were not able to establish any association between the presence of lesions and either malabsorption, weight loss, or gastric bile acid concentrations, we did notice that OAGB rats with a long BPL experienced the greatest malabsorption and highest incidence of esogastric lesions.

Patients that undergo bariatric surgery are fairly young with a mean age of 42^31^. Based on the WHO’s Global Health Observatory data in 2016, these patients are expected to live another 30 years with worldwide life expectancy averaging 72 years^32^. As such, they are at risk for long-term side effects that could arise as the result of these procedures. This preclinical OAGB study presents a longitudinal study with a 30 weeks follow-up in rats that is equivalent to 22–30 years of human life^33^. In order to maintain clinical relevance, we compared OAGB to the gold standard RYGB, and we used rats that did not undergo any operation as a control group to recapitulate patients without bariatric surgery.

As a result of the anatomical remodeling of the gastric pouch, risks of biliary reflux and resulting long-term complications still leave OAGB a controversial procedure today. Our study provides evidence that the risk of esophageal cancer after OAGB remains a possibility after observing FH and esophagitis lesions. Even though FH is a sign of chemical gastritis and cannot be strictly interpreted as precancerous lesions, physiological mechanisms leading to FH could be similar to those leading to esophageal lesions. The presence of these lesions signify the earliest step of the esophageal carcinogenic sequence. Involvement of BA is suggested by the occurrence of FH at about 60% in the OAGB groups compared to 10% in RYGB group. The complex role of biliary reflux as either an unassociated independent factor or as a potentializing factor of pre-existent gastro-esophageal reflux (GER) has been suggested by several studies^14,34^. Yet, in our present study, residual gastric pouch BA concentrations were not associated with the occurrence of anatomopathological lesions. This interesting finding suggests that BA exposure to esophageal mucosa may not by itself promote esogastric anatomopathological lesions. Furthermore, a longer BPL was associated with an increased rate of esophageal lesions compared to a shorter BPL despite a trend toward lower gastric pouch BA concentrations in the long BPL group. This observation runs against the notion that a longer BPL could decrease the risk of esophagitis due to partial reabsorption of BA^35^. We hypothesize that the nutritional status, notably undernutrition, could be a co-factor in esophageal lesions that incite carcinogenesis. It is well known that gastric bypass may lead to micronutrient deficiencies^28,29,36^. OAGB is suspected to be associated with an increased sarcopenic undernutrition^37^, and a longer BPL is known to increase nutritional complication and undernutrition^28,38^. Accordingly, the OAGB rats with the long BLP experienced a drastic initial weight loss, a concurrent lower weight gain at the end of the experiment and reduced vitamin D plasma levels. OAGB rats with the long BLP were also associated with the highest occurrence of esophagus lesions. Undernutrition and micronutrient deficiencies could thus weaken the esophageal mucosa against aggressions secondary to biliary reflux, overpowering the beneficial features of OAGB and increasing the chances for carcinogenesis.

A paradoxical observation we reported was the association of reduced weigh loss with esophageal hyperpapillomatosis but not with esophagitis. One could hypothesize this to be a physiological adaptation promoting nutrient absorption in food-depleted animals as already reported in Short Bowel Syndrome (SBS) animal models^39^. However, in the SBS model of undernutrition, hyperplasia has been described in the colon and jejunum, whereas the esophagus was not studied. Transposition of jejunum within the esophagus has been reported to induce hyperplasia of the transposed jejunum^40^. Nevertheless, to the best of our knowledge, study of the adaptation of the esophagus (hyperplasia and/or hyperpapillomatosis) and its contribution to nutrient absorption in undernourished animal models has never been published and merit further attention.

Despite the fact that long-term safety of OAGB has not been unequivocally established, it appears that specific characteristics of OAGB and RYGB could protect the esophageal mucosa against biliary reflux consequences. Rats are recognized as a good model of esophageal carcinogenesis and a promising model to reproduce the human carcinogenic sequence^13,41^. In other experimental studies, esojejunal anastomosis on rats led to Barrett’s esophagus in 92% of cases after 30 weeks and cancer rate up to 8% without any co-carcinogen in F344 rats^42^ and esophageal intestinal metaplasia and mucosal ulcerations were observed in 41.7% and 50% of wistar rats with esojejunal anastomosis^43^. These experiments and ours suggest putative protective factors specific to gastric bypass surgeries^43^. GER is a well-known risk factor of esophageal cancer^34,44^. Here, we propose the potential impact of GER on esophageal mucosa because unoperated rats presented a rate of esophageal hyperpapillomatosis (EHP) of 50%, alleging that quadruped animals might be exposed to a physiological GER. In contrast, EHP lesions were almost absent in the RYGB group, revealing an inherent protective effect of a gastro-jejunal anastomosis on the esophageal mucosa. The gastro-jejunal anastomosis bypasses the pylorus, which likely leads to a reduction of acid production and gastric pressure. This hypothesis is supported by the positive results of the RYGB on GER disease symptoms and preservation of esophageal function in humans^45^. This supports why RYGB is considered a therapeutic option in the case of resistant GER disease^46,47^. As for OAGB surgery, acidic gastric secretions produced in the gastric pouch could be neutralized by basic BA, reducing their agressivity^13,48^. In humans, but not in our experimental model, the OAGB’s long and tubular gastric pouch should mechanically diminish exposure of the esophagus to gastric content in contrast to the small RYGB gastric pouch. In addition, weight loss post-bariatric surgery leads to a reduction of GER^49^. Altogether, these factors could explain why there is no case of esophageal cancer described in this experiment and why only one case was reported 20 years after the first human OAGB^21^.

We recognize that our study focuses on the anatomy of rats and still requires further investigation in order to be translated to human physiology. Rat esophageal histology is inherently different from humans due to the presence of a keratinized epithelium, not to mention quadrupeds naturally display esophageal and gastric lesions. In addition, the limited sample size resulted in a low power of most of our statistical analyses and prevented us from exploring whether there was an additive effect of BA and undernutrition in regards to the development of esogastric lesions. We must also note that unoperated animals as a baseline control may yield slightly different histological physiology than those that would have underwent a sham surgery. However, we found that this would most closely mirror patients without surgery. Our model of lean Wistar rats must also be taken into consideration when studying carcinogenesis, since obesity is a well-known risk factor of cancer^50^, especially esophageal cancer^51^. Nevertheless, this experimental design on lean animals allowed us to study individual procedure impact independent of obesity, preventing weight loss as a cofounding factor and revealing baseline effects of bariatric surgery on gastrointestinal physiology. Observing subtle changes in lean rats provides compelling concerns that biliary reflux could have an additive effect in cancer risk when coupled with obesity, and definitive conclusions would require further investigation in obese rats.

Most importantly, a clinical study exploring esogastric mucosa longitudinally after OAGB is needed to confidently eliminate the risk of cancer following this procedure. The recent finding that sleeve gastrectomy has been associated with a higher risk of Barrett’s esophagus^52^ provides a compelling reason for repeated endoscopic exploration after surgery^53^. Until longitudinal endoscopic studies post-surgery are conducted to characterize the negative impact of OAGB on esogastric mucosa, endoscopic follow-up is crucial in determining whether or not carcinogenicity can be ruled out.

## Materials and Methods

### Ethics

All animal studies comply with the ARRIVE guidelines. They were conducted in accordance with EU directive 2010/63/EU for animal experiments and approved by the Institutional Animal Care and Use Committee (CEA N° 121) and the French Ministry of Higher Education and Research (APAFIS #02285.03).

### Preclinical surgical model

After a week of habituation, 48 Wistar rats (7 weeks old) were randomly assigned to a unoperated group (CTRL n = 8), or to RYGB (n = 13), OAGB-15 (n = 14) and OAGB-35 (n = 13) surgery.

Surgical models have already been described in detail in previous publications^37,54^ and are only briefly described below.

Gastric pouch construction was similar in OAGB and RYGB. After laparotomy, loose gastric connections to the liver and the spleen were released along the greater curvature, and the suspensory ligament supporting the upper fundus was severed. A 35 mm staple gun was applied at the junction between the glandular and non-glandular stomach to the left allowing resection of the forestomach. A second stapler using a thoraco-abdominal device (3–3.5 mm) was applied, parallel to the first stapling, on the right side of the gastroesophageal junction to exclude the antrum and a part of the body of the stomach. The two staple lines result in a ~0.5 cm wide gastric pouch.

For rats operated on OAGB, the jejunum was anastomosed to the gastric pouch 15 cm (OAGB-15) or 35 cm (OAGB-35) from the duodenojejunal angle with 7-0 isotactic polypropylene running sutures.

For rats operated on RYGB, the jejunum was transected 15 cm distally from the duodenojejunal angle. The Roux limb was anastomosed to the gastric pouch and the biliopancreatic limb was anastomosed 20 cm distal to the gastrojejunal anastomosis with 7-0 isotactic polypropylene running sutures.

The limb lengths chosen for the RYGB and OAGB-35 reproduce the ratio between the different segments commonly done in human procedures. OAGB-15 corresponds to an OAGB with a short BPL, inferior to 1 m, in humans.

All rats were kept with free access to water during day 1 post-surgery. On day 2 and 3 post-surgery, rats had free access to a liquid diet, and on day 4, they had access ad libitum to a normal diet. Unoperated rats followed the same post-operative nutrition protocol.

The overall survival rate was 82% with death (9/48) occurring in the immediate post-operative period. Out of the 9 deaths, 3 occurred in each group of gastric bypass.

### Bile acid concentrations

After euthanasia, esophagi and stomachs were removed and flushed with 500 µL of phosphate buffered saline (D-PBS) that were collected and stored at −20 °C. Bile acids were extracted by solid-phase extraction and analyzed using high-performance liquid chromatography tandem mass spectrometry (HPLC MS/MS) as previously described^55^.

### Calorimetric analyses

Macronutrient absorption was determined by fecal analyses as previously described^37^. Stools were collected within 24 h during the second postoperative week. Quantification of daily energetic loss (kCal/24 h) was determined by bomb calorimetry (PARR 1351 Bomb Calorimeter; Parr Instrument) and the daily caloric loss (%) was expressed as a ratio of daily energetic loss on daily food intake.

### Biochemical analyses

Blood samples (plasma) were collected during euthanasia 30 weeks after surgery. Samples were used for determination of concentrations of creatinine, urea, albumin, protein, calcium, phosphates, iron, ferritin, transferrin, triglycerides, total and HDL cholesterol, Non Esterified Fatty Acids (NEFA), Alanine Aminotransferase (ALAT) and Aspartate Aminotransferase (ASAT), using an automatic analyzer AU400 (Olympus Diagnostics, Rungis, France). Vitamin D was assayed, with IDS-iSYS 25 VitDS (Immunodiagnostic Systems Holdings PLC, UK) assay following manufacturer’s instructions.

### Histological analyses

After euthanasia, routine histology i.e. Hematoxylin-Eosin-Saffron (HES) and Alcian Blue-Periodic Acid Schiff (AB-PAS) were performed on formalin-fixed esophagus and gastro-jejunal anastomosis.

Parameters analyzed were the presence of dysplasia, metaplasia, and cancer. Presence of esophageal hyperpapillomatosis (EHP) and esophagitis as well as foveolar hyperplasia (FH) on gastric mucosa, a feature of reactive gastritis, were also studied. Esophagitis was defined by the association of basal cell hyperproliferation, fibrosis, hyper ortho-keratosis, immune cell infiltration and presence of EHP. FH was defined as the association of fibrosis, hyperproliferation of smooth muscle cells, and parietal cells. Slides were interpreted by two expert anatomopathologists (AC and MH) blinded for the corresponding animal group.

### Immunohistochemistry

MUC2, MUC5B and MUC4 protein expression was studied using an automated 8 immunostainer (ES, Ventana Medical System, France) with the following antibodies MUC2 (H300 rabbit, sc15334 Santa Cruz 1/200), MUC5B (mouse, EUMUC5B 1/50), and MUC4 (1G8 mouse, sc33654 Santa Cruz 1/50). Positive controls were included by staining normal rat tissues known to express a protein of interest, and negative controls were run with D-PBS instead of primary antibodies.

### Statistical analyses

Quantitative values are expressed as mean ± SEM. Comparison between groups was conducted using ANOVA after log conversion or Kruskal Wallis non-parametric tests followed by Dunn’s multiple comparison tests where appropriate. Kinetic studies were analyzed with a 2-way ANOVA followed by Tukey’s multiple comparison tests where appropriate. Qualitative values were compared using Fisher exact test. Empirical power at the 5% level has been obtained by Monte-Carlo simulations using 500 replications. For contingency table, the observed table was used to estimate the proportion in each group. For quantitative data, a linear model adjusted on the observed data was used to obtain the mean by factor and the standard deviation.

All analyses were conducted with R version 3.6.1 (2019-07-05) or GraphPad prism 7.03.

*P* < 0.05 was considered to be significant.

## Supplementary information

Supplementary Figures.
