# Supplementary material for: Long-term consequences of one anastomosis gastric bypass on esogastric mucosa in a preclinical rat model
Source: Sci Rep. 2020 Apr 30;10:7393. doi: 10.1038/s41598-020-64425-2 (PMC7192900; doi:10.1038/s41598-020-64425-2)
Supplement: Supplementary file 1 — Supplementary Figures. [file 41598_2020_64425_MOESM1_ESM.pdf]

Long-term consequences of one anastomosis gastric bypass on esogastric mucosa in a preclinical rat model

Matthieu Siebert, MD[1,2], Lara Ribeiro-Parenti, MD, PhD[1,3], Nicholas D. Nguyen[1], Muriel Hourseau, MD[4], Belinda Duchêne[5], Lydie Humbert[6], Nicolas Jonckheere, PhD[5], Grégory Nuel, PhD[7], Jean-Marc Chevallier, MD, PhD[2], Henri Duboc, MD, PhD[1], Dominique Rainteau, PhD[6], Simon Msika, MD, PhD[1,3], Nathalie Kapel MD, PhD[8], Anne Couvelard, MD, PhD[4], André Bado, PhD[1], Maude Le Gall, PhD[1,*]

[1]Inserm UMRS 1149, UFR de Médecine Paris Diderot, Université de Paris, AP-HP, Paris, France

[2]Department of digestive Surgery, AP-HP, Hôpital Européen Georges Pompidou, Paris, France

[3]Department of General and Visceral Surgery, AP-HP, Bichat-Claude Bernard Hospital, Paris, France

[4]Department of anatomopathology, AP-HP, Bichat-Claude Bernard Hospital, Paris, France

[5] Univ. Lille, CNRS, Inserm, CHU Lille, UMR9020 – UMR-S 1277 - Canther – Cancer Heterogeneity, Plasticity and Resistance to Therapies, Lille, France.

[6]Inserm UMR 7203, AP-HP Saint Antoine hospital, Paris, France

[7] Stochastics and Biology Group (MAV), Probability and Statistics (LPSM), CNRS 8001, Sorbonne Université, Paris, France.

[8] Laboratoire de Coprologie Fonctionnelle, Hôpital Pitié-Salpêtrière Charles Foix, AP-HP, Paris, France.

***correspondence to:**

Maude Le Gall, PhD,

Maude.le-gall@inserm.fr

Tel: +33 (0)157 277 459, Fax: +33 (0)157 277 471

Short running title

OAGB and esogastric mucosa

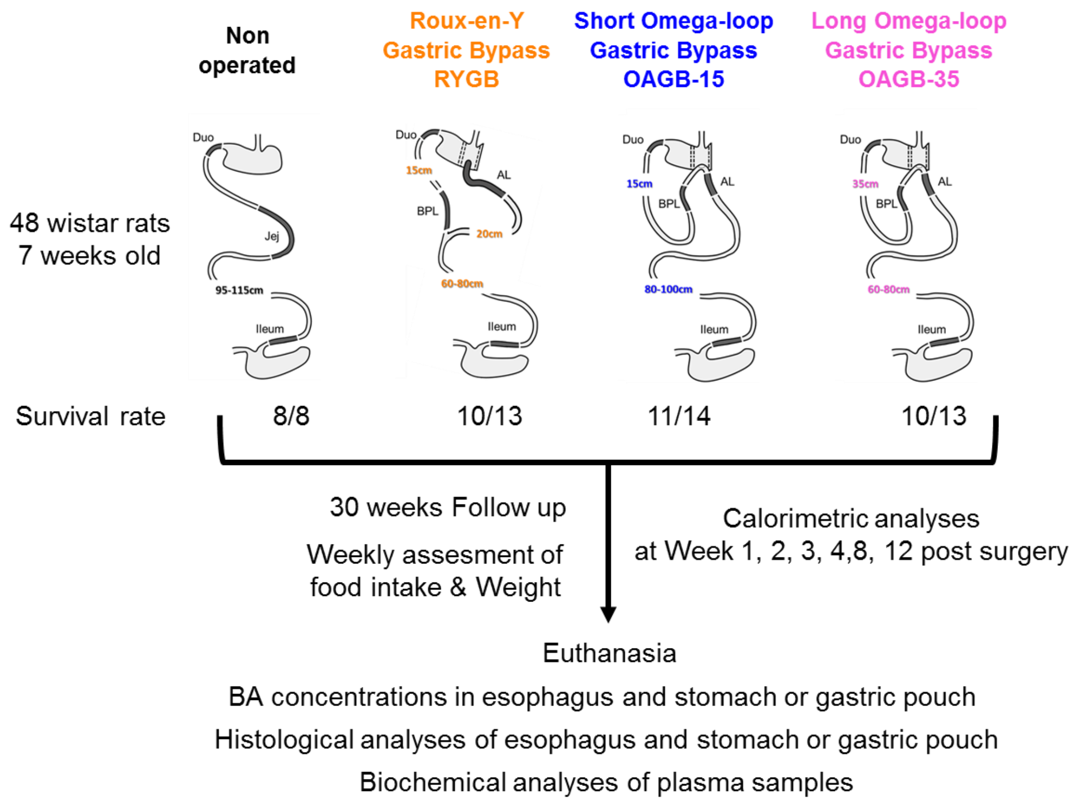

SUPPLEMENTARY FIGURE S1 General Organization of the study

48 Wistar rats (7 weeks old) were randomly assigned to an unoperated group (CTRL n = 8), or to RYGB (n = 13), OAGB-15 (n = 14) and OAGB-35 (n = 13) surgery.
Food intake and weight were assayed weekly and a 24h calorimetric analysis was performed at week 1, 2, 3, 4, 8, and 12 post-surgery. After 30 weeks, rats were euthanized, blood and esogastric tissues were sampled. BA concentrations were measured in the esophagus and gastric pouch, histological analyses were performed on esophagus and gastric mucosa, concentrations of numerous biological parameters were determined in the blood.

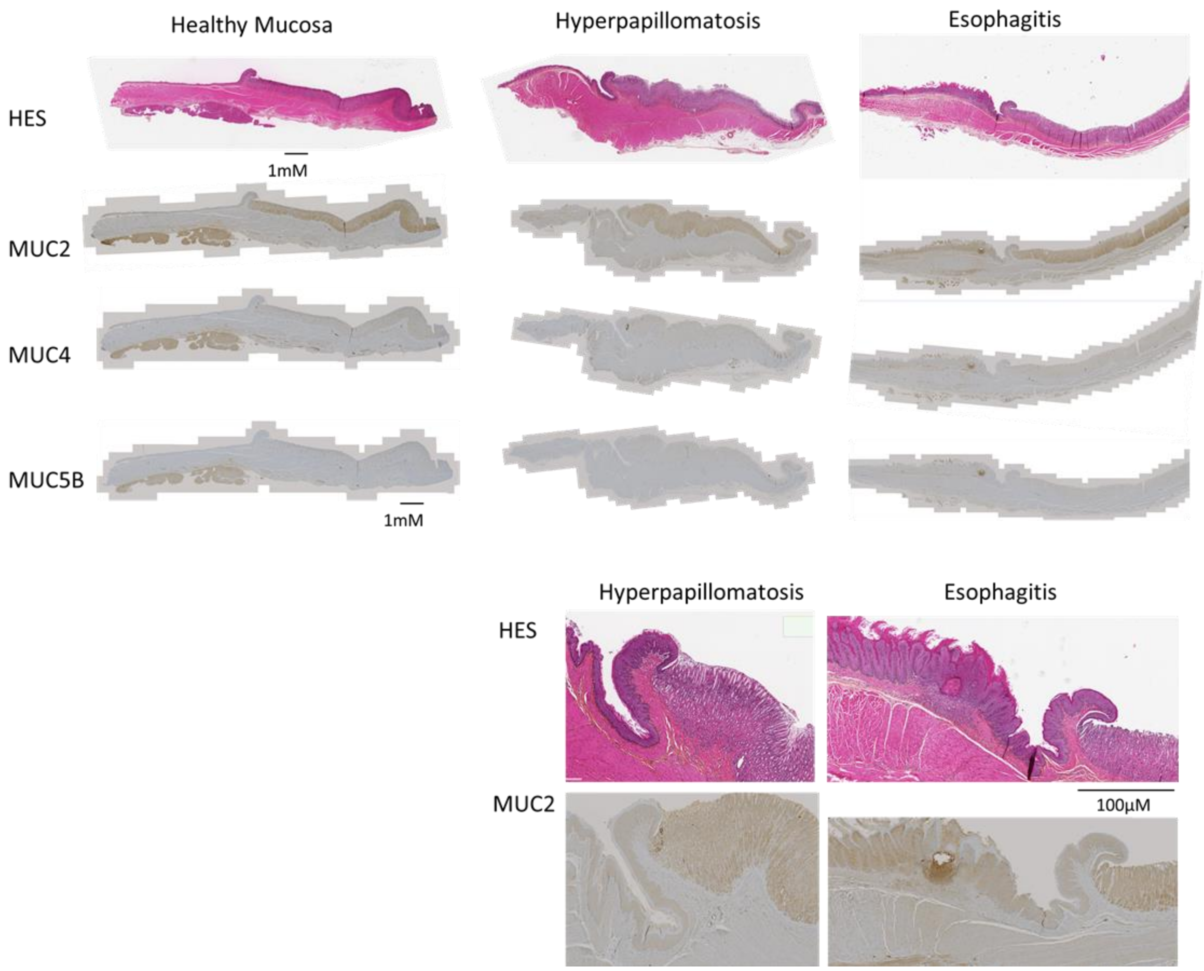

SUPPLEMENTARY FIGURE S2 Gastrointestinal lesions are not associated with Barrett's esophagus

Representative HES staining and MUC2, MUC4 and MUC5B immunostaining of healthy esophageal mucosa, esophageal hyperpapillomatosis and esophagitis
Note the absence of expression of Barrett's esophagus markers.

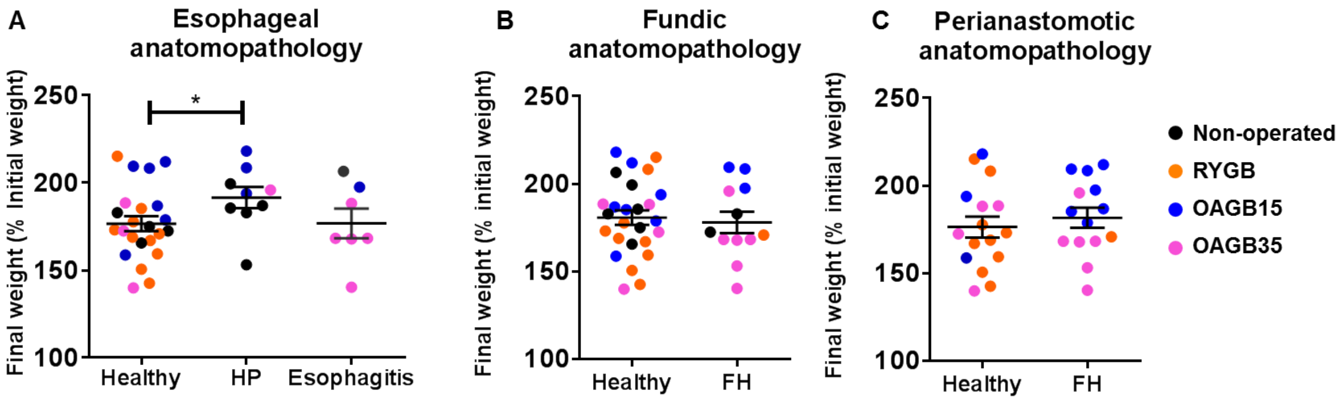

SUPPLEMENTARY FIGURE S3 : Final weight 30 weeks post-surgery are not associated with esogastric pathologies

Esophageal (A), fundic (B) and perianastomotic (C) anatomopathology according to final weight loss.

(Sham in black, RYGB in orange, OAGB-15 in blue, OAGB-35 in pink).

* $P < 0.05$ by Dunn’s multiple comparison tests after Kruskal Wallis test.

	HEM	EHP	Esophagitis
CTRL	4	3	1
RYGB	9	1	0
OAGB-15	6	3	1
OAGB-35	3	1	5

Supplementary Table 1 : Original data and statistical analyses of Esophageal anatomopatholy Fisher exact test p-value 0.046.

	HM	FH
CTRL	6	2
RYGB	9	1
OAGB-15	7	3
OAGB-35	4	6

Supplementary Table 2 : Original data and statistical analyses of Fundic anatomopatholy. Fisher exact test p-value 0.13.

	HM	FH
RYGB	9	1
OAGB-	3	7
OAGB-	4	6

Supplementary Table 3: Original data and statistical analyses of Perianastomotic anatomopatholy. Fisher exact test p-value 0.019.

	power1 (5%)	power2 (5%)	power1 (1%)	power2 (1%)
Table1	0.776	0.994	0.572	0.962
Table2	0.508	0.876	0.268	0.662
Table3	0.800	0.978	0.548	0.928

Supplementary Table 4: Power analysis for the qualitative tests (Fisher exact test) at level 5% (left) and 1% (right). power1 correspond to the actual sample size, power2 to the doubled sample size.

	power1 (5%)	power2 (5%)	power1 (1%)	power2 (1%)
Total BA	0.642	0.942	0.364	0.820
Primary BA	0.688	0.974	0.432	0.884
Secondary BA	0.332	0.644	0.130	0.382

Supplementary Table 5: Power analysis for the quantitative tests (one-way anova) of the BA concentration at level 5% (left) and 1% (right). power1 correspond to the actual sample size, power2 to the doubled sample size.

SUPPLEMENTARY TEXT 1

Additional statistical analyses on esophageal and gastric lesions conducted with R version 3.6.1 (2019-07-05).

Control analyses: CTRL *vs* RYGB

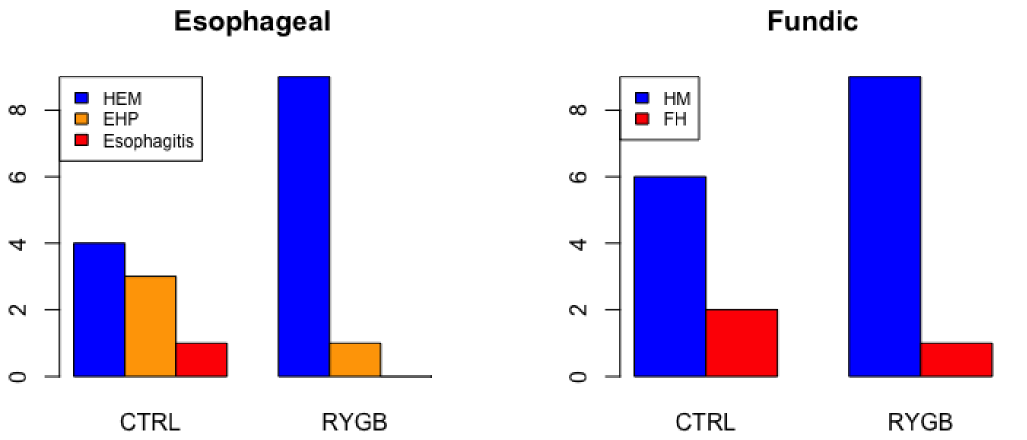

Fisher exact test p-value Left: 0.157. Right: 0.559. Both combined: 0.368.
From this figure, we conclude that there is no significant difference between NO and RYGB. The strongest significance level was 0.157 (left panel). With the study design, the power (at level 5%) for the corresponding table is 0.352 which is low. The non-significant might here be due to the low power of the study. Further investigation with greater power could be more informative to determine whether RYGB may meliorate spontaneously occurring esophageal lesions.

Primary analyses: RYGB vs OAGB-15 vs OAGB-35

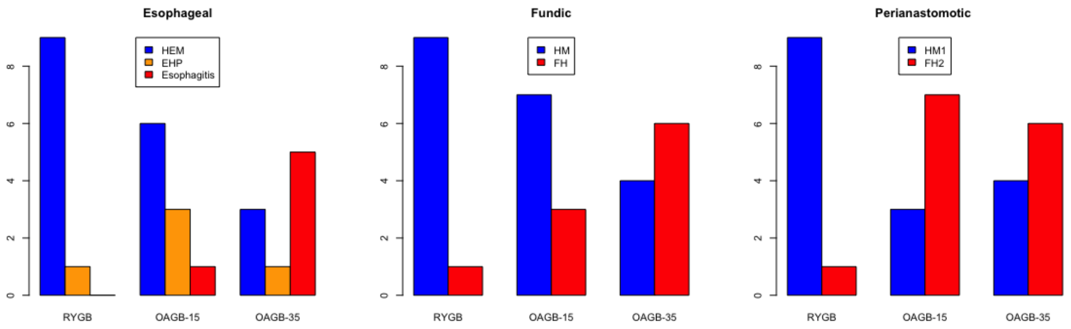

Fisher exact test p-value Left: 0.0184. *Middle:* 0.08. *Right:* 0.0191.

Secondary analyses: RYGB vs OAGB-15

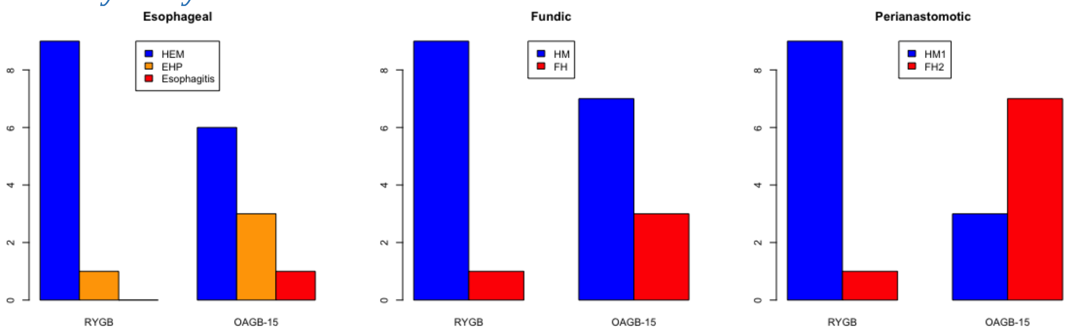

Fisher exact test p-value Left: 0.303. *Middle:* 0.582. *Right:* 0.0198.

Secondary analyses: RYGB vs OAGB-35

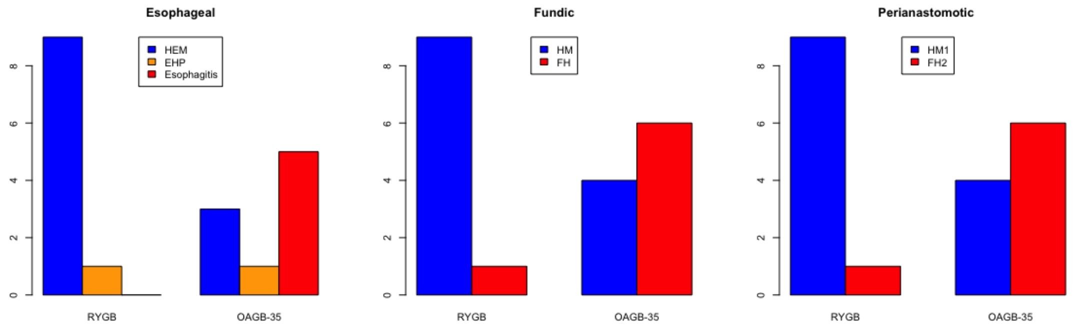

Fisher exact test p-value Left: 0.00786. *Middle:* 0.0573. *Right:* 0.0573.
Note that two p-values for the qualitative comparison of RYGB *vs* OAGB-35 (Fundic and Perianastomotic) are non-significant but very close to the threshold of 5%.
The corresponding power is 0.436 for Fundic and 0.444 for Perianastomotic.
If we combine both tables, we obtain a p-value of 0.0137.

Secondary analyses: OAGB-15 vs OAGB-35

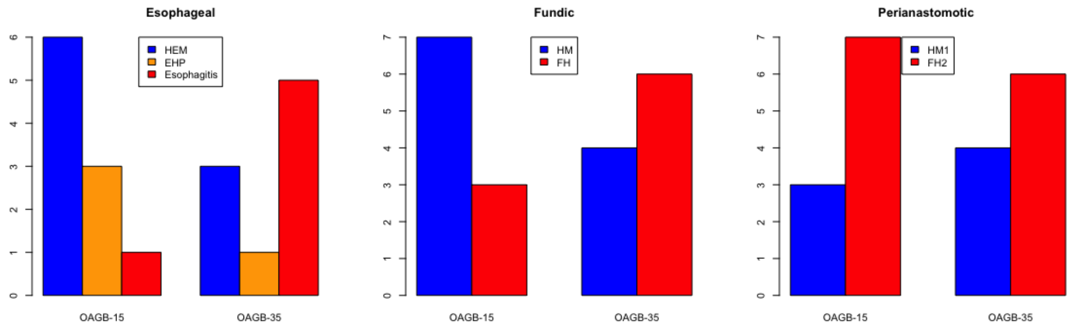

Fisher exact test p-value Left: 0.143. *Middle:* 0.37. *Right:* 1.

SUPPLEMENTARY TEXT 2

Additional statistical analyses on BA concentrations conducted with R version 3.6.1 (2019-07-05).

Control analyses: CTRL *vs* RYGB

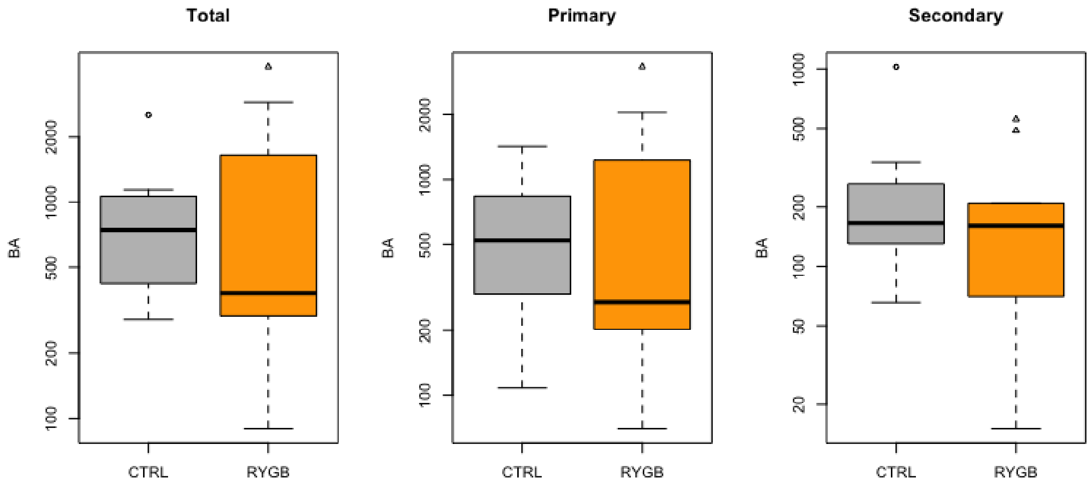

one-way anova p-value Left: 0.649. *Middle:* 0.779. *Right:* 0.342.

We conclude that there is no significant difference between CTRL and RYGB.

Primary analyses: RYGB *vs* OAGB-15 *vs* OAGB-35

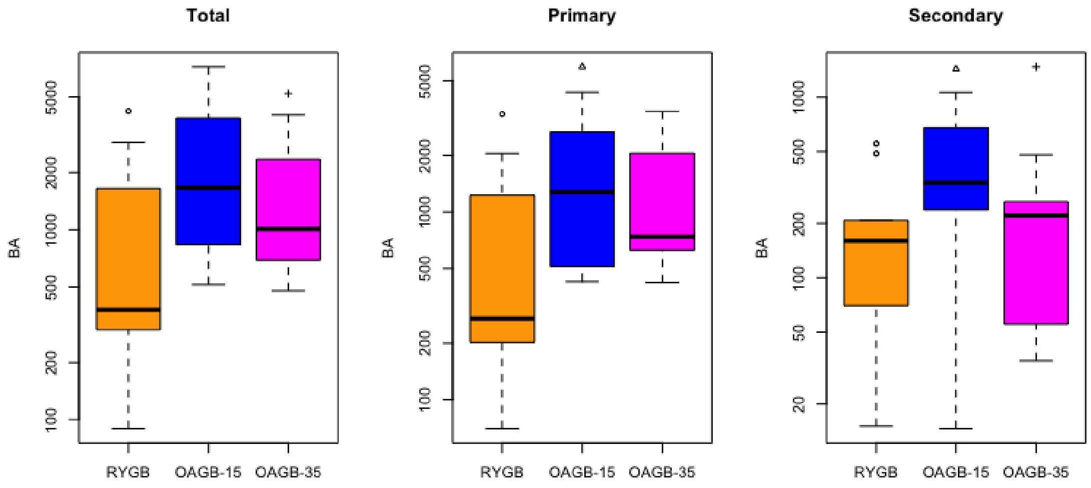

one-way anova p-value Left: 0.0504. *Middle:* 0.0375. *Right:* 0.179.

There is clearly a significant difference between the three surgeries.

For the third dataset, the p-value is 0.179 and the power at level 5% is 0.356, thus the unsignificant results is probably due to the low power of the design.

Secondary analyses: RYGB vs OAGB-15

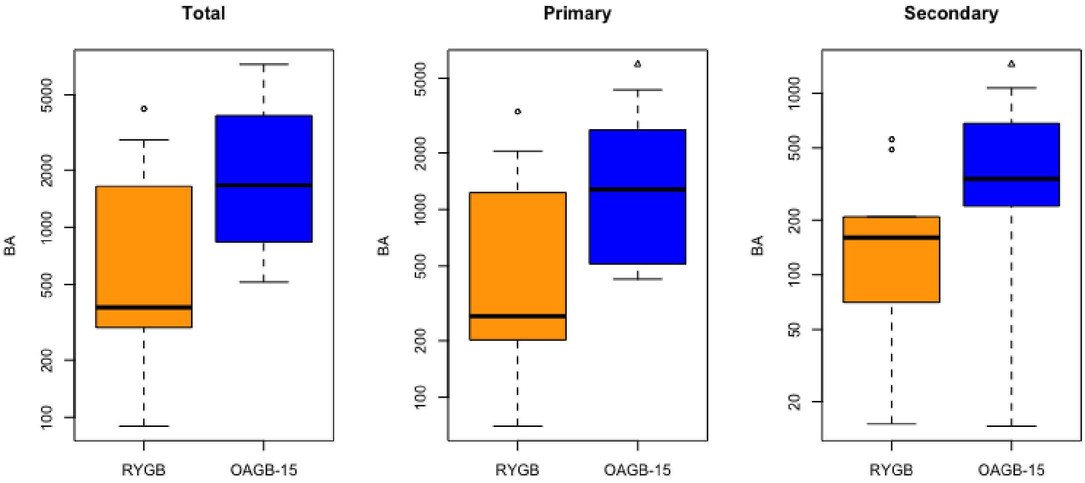

one-way anova p-value Left: 0.0279. *Middle:* 0.0271. *Right:* 0.0717.

Secondary analyses: RYGB vs OAGB-35

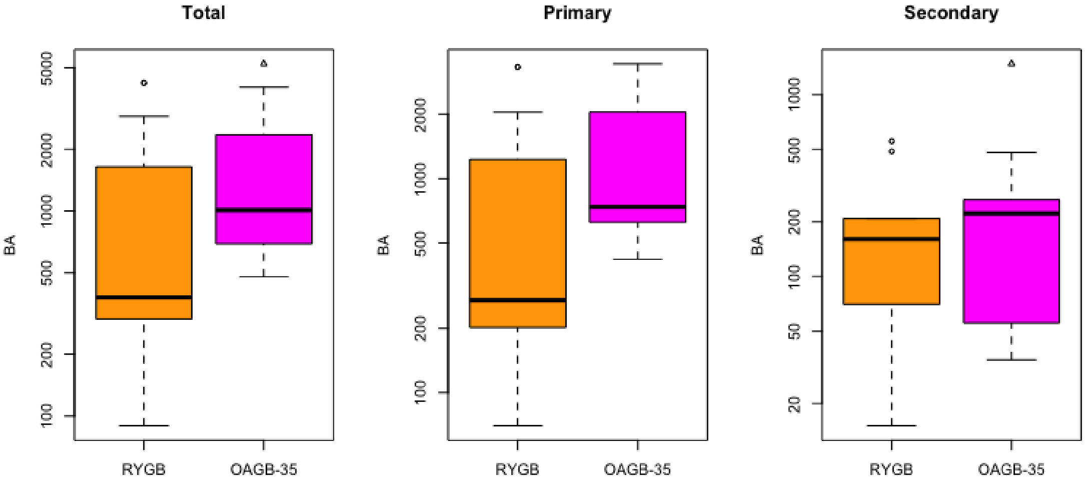

one-way anova p-value Left: 0.108. *Middle:* 0.0636. *Right:* 0.563.

Secondary analyses: OAGB-15 vs OAGB-35

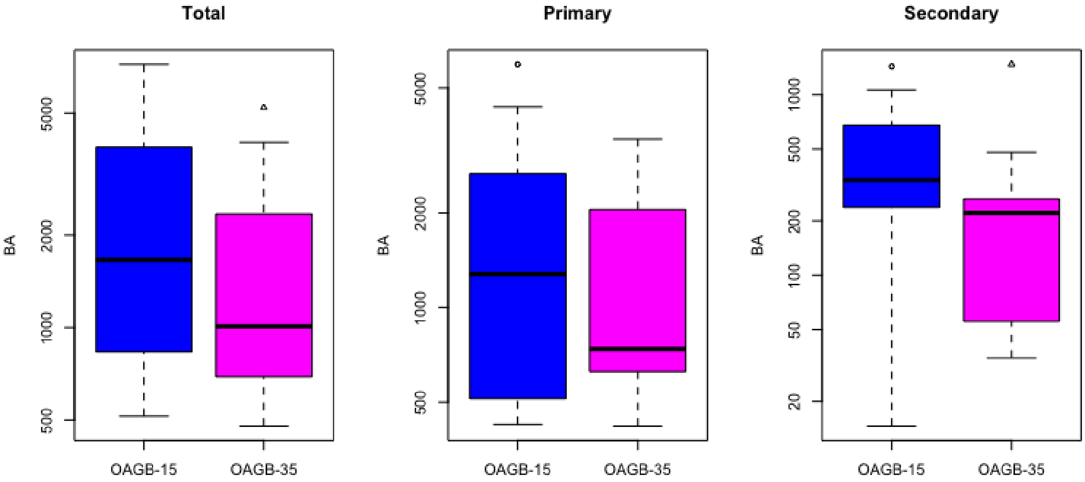

one-way anova p-value Left: 0.486. *Middle:* 0.65. *Right:* 0.249.
